# FAM20A Deficiency Drives Transcriptomic Dysregulation and Functional Impairment in Gingival Fibroblasts

**DOI:** 10.1111/cpr.70096

**Published:** 2025-07-22

**Authors:** Kanokwan Sriwattanapong, Sermporn Thaweesapphithak, Chompak Khamwachirapitak, Pannagorn Sae‐ear, Sasiprapa Prommanee, Noppadol Sa‐Ard‐Iam, Suphalak Phothichailert, Han Sung Jung, Vorasuk Shotelersuk, Thantrira Porntaveetus

**Affiliations:** ^1^ Center of Excellence in Precision Medicine and Digital Health, Center of Excellence in Genomics and Precision Dentistry, Department of Physiology, Faculty of Dentistry Chulalongkorn University Bangkok Thailand; ^2^ Oral Biology Research Center, Faculty of Dentistry Chulalongkorn University Bangkok Thailand; ^3^ Clinical Research Center, Faculty of Dentistry Chulalongkorn University Bangkok Thailand; ^4^ Center of Excellence in Periodontal Disease and Dental Implant, Immunology Research Center, Faculty of Dentistry Chulalongkorn University Bangkok Thailand; ^5^ Center of Excellence for Dental Stem Cell Biology, Faculty of Dentistry Chulalongkorn University Bangkok Thailand; ^6^ Department of Oral Biology Yonsei University College of Dentistry Seoul Republic of Korea; ^7^ Center of Excellence for Medical Genomics, Department of Pediatrics, Faculty of Medicine Chulalongkorn University Bangkok Thailand; ^8^ Excellence Center for Genomics and Precision Medicine King Chulalongkorn Memorial Hospital, The Thai Red Cross Society Bangkok Thailand; ^9^ Graduate Program in Geriatric and Special Patients Care, Faculty of Dentistry Chulalongkorn University Bangkok Thailand; ^10^ Clinic of General, Special Care and Geriatric Dentistry, Center for Dental Medicine University of Zurich Zurich Switzerland

**Keywords:** diagnosis, healthcare, renal stones, stem cells, therapy, tooth eruption

## Abstract

Amelogenesis imperfecta type 1G (AI1G), also known as Enamel‐Renal‐Gingival Syndrome (ERGS), is an autosomal recessive disorder caused by variants in *FAM20A*, encoding a Golgi apparatus protein crucial for protein processing and secretion. AI1G presents with enamel defects, nephrocalcinosis and gingival overgrowth. Building upon our previous findings demonstrating the impact of FAM20A insufficiency on deciduous dental pulp cells, this study investigated the molecular mechanisms underlying gingival fibromatosis in AI1G. RNA sequencing of gingival fibroblasts from an AI1G patient revealed widespread differential gene expression (DEG). Gene Ontology (GO) analysis demonstrated enrichment of DEGs in biological processes related to cell adhesion, differentiation, proliferation (including positive regulation and cell division), cell cycle regulation, apoptosis and signal transduction. Pathway analysis (Reactome and KEGG) further highlighted the dysregulation of signalling pathways, including Wnt, TGF‐β, cell cycle, DNA replication, Rho GTPase signalling and extracellular matrix organisation. Functional assays confirmed these findings, revealing delayed initial attachment and spreading, impaired osteogenic differentiation (evidenced by reduced mineralization and downregulation of *DLX5*, *OCN*, *RUNX2* and *OPN*), enhanced cell cycle progression and proliferation (increased colony size and proliferation rates, along with a shift from G0/G1 to G2/M phase) and suppressed apoptosis in FAM20A‐insufficient fibroblasts. These results suggest that FAM20A plays a critical role in regulating fundamental processes in gingival fibroblasts, and its insufficiency contributes to the gingival fibromatosis phenotype observed in AI1G through the disruption of cell adhesion, differentiation, proliferation and apoptosis. This study proposes novel insights into the pathogenesis of AI1G and highlights potential therapeutic targets for this complex disorder.

## Graphical abstract

*FAM20A* variants cause AI1G, marked by enamel defects, gingival overgrowth and ectopic calcifications. RNA sequencing of patient‐derived gingival fibroblasts showed dysregulated genes in adhesion, proliferation and signalling pathways. Functional assays revealed increased cell proliferation, impaired ECM interactions and osteogenesis, suggesting FAM20A dysfunction disrupts tissue remodelling and contributes to tooth eruption failure.

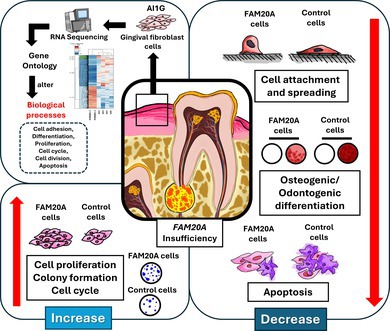

## 1 Introduction

Amelogenesis imperfecta (AI) encompasses a group of genetic disorders that disrupt enamel development, resulting in a spectrum of dental defects including discoloration, hypoplasia, hypomineralisation and hypomaturation [1, 2]. These defects can lead to increased tooth sensitivity, aesthetic concerns and impaired dental function. While significant progress has been made in identifying the genes implicated in AI, the complex cellular and molecular mechanisms underlying this condition remain incompletely understood. Among these, *FAM20A* (family with sequence similarity 20, member A; OMIM *611062) emerges as a particularly intriguing factor. Biallelic loss‐of‐function variants in *FAM20A* are causative of a rare subtype, amelogenesis imperfecta type 1G (AI1G), also known as Enamel‐Renal‐Gingival Syndrome (ERGS) [3, 4, 5, 6, 7]. This syndrome is characterised by a distinctive clinical triad: defective enamel formation, gingival hyperplasia and renal anomalies. Investigating the role of *FAM20A* in these interconnected developmental processes has the potential to shed light on the pathogenesis of AI1G and advance our understanding of the fundamental mechanisms regulating biomineralisation and tissue homeostasis.

FAM20A, a Golgi‐resident protein kinase, plays a crucial role in protein processing and secretion. Previous research has demonstrated that reduced FAM20A levels in deciduous dental pulp cells (DDP) impact fundamental cellular processes such as proliferation, migration, and attachment and dysregulate osteogenic differentiation and inflammatory responses [8]. These findings underscore the importance of FAM20A for proper DDP function and implicate its dysfunction in the pathogenesis of AI1G. Notably, gingival overgrowth, a characteristic feature of AI1G, suggests a role for FAM20A in regulating gingival fibroblast activity, the principal cellular component of periodontal tissue [9, 10]. However, the precise mechanisms by which *FAM20A* variants affect gingival fibroblasts remain poorly understood. It is hypothesized that FAM20A dysfunction may impair the production or secretion of growth factors and signalling molecules, leading to altered fibroblast proliferation and differentiation.

This study utilised gingival fibroblasts as a model system to investigate the pathomechanisms underlying FAM20A‐associated gingival fibromatosis and other potential biological consequences of AI1G. Through transcriptomic analysis and functional assays, we aimed to elucidate the cellular and molecular alterations induced by *FAM20A* variants in these cells. This work sought to bridge the gap in understanding how a single gene variant, *FAM20A*, could result in seemingly disparate phenotypes, including soft tissue overgrowth (gingival fibromatosis) and hard tissue hypoplasia (enamel defects), ultimately contributing to the development of novel therapeutic strategies for AI1G.

## 2 Material and Methods

### 2.1 Samples

Gingival fibroblast cells were isolated from the gingival tissue of a patient diagnosed with AI1G, harbouring the previously reported compound heterozygous variants, c.1231C>T and c.1109+3_1109+7delinsTGGTC in *FAM20A* [6, 11]. These cells, referred to as FAM20A cells in this study, were compared with gingival fibroblasts from six age‐ and sex‐matched Thai healthy individuals without systemic diseases or orodental abnormalities (controls). Our previous study demonstrated that FAM20A gingival fibroblast cells exhibited a significant reduction in both *FAM20A* mRNA and protein expression levels [6, 11].

### 2.2 Cell Isolation and Culture

The gingival tissues were cut into 1 × 1 mm pieces and placed in a 35‐mm culture dish (Corning, New York, USA). Cells were maintained in growth medium consisting of Dulbecco's Modified Eagle Medium (DMEM), 10% fetal bovine serum (Gibco, CA, USA), 1% ‐glutamine (Gibco, CA, USA), and 1% antibiotic‐antimycotic (Gibco, CA, USA), and incubated in a humidified environment at 37°C and 5% CO_2_. Cells from passages 5–7 were used in the experiments. In total, we included six biological replicates from healthy controls. Four of these (GF1–GF4) were used for RNA sequencing to capture biological variation, while the remaining two control samples (Cont1 and Cont2) were used for validation experiments, including qPCR and functional assays. For the FAM20A group, due to the rarity of AI1G, only one patient sample was available. From this, we generated two experimental replicates (FAM20A‐1 and FAM20A‐2) by isolating fibroblasts from different gingival tissue into separate culture dishes and treating them as independent samples for RNA sequencing. A similar strategy has been employed in other FAM20A‐related studies, where, due to the rarity of the disease, tissue samples from a single patient were used [8, 10, 12, 13]. In the qPCR and functional assays, we used FAM20A‐derived cells and Cont1 and Cont2, and for each of these, we performed at least three experimental replicates to ensure statistical reliability. These experimental replicates were independently processed and analysed to allow appropriate statistical testing.

### 2.3 RNA Preparation and Sequencing

RNA was extracted using the RNeasy Mini Plus Kit (QIAGEN, CA, USA) following the manufacturer's protocol, including DNase I treatment. The integrity of total RNA was assessed using a Bioanalyzer 2100 (Agilent 2100, CA, USA), with a minimum RNA Integrity Number (RIN) value of 7 accepted for analysis. RNA sequencing was conducted using the Illumina NovaSeq6000 platform (TruSeq stranded mRNA library) in 100 bp paired‐end run mode, generating 40 million reads per sample (Macrogen Inc., Seoul, Korea).

### 2.4 RNA Sequencing Analysis

The raw sequencing data (fastq) were aligned to the *Homo sapiens* reference genome (UCSC hg19) using the RNA‐Seq Alignment program (Basespace, Illumina, CA, USA). Differential gene expressions were analysed using RNA‐Seq Differential Expression (Basespace, Illumina, CA, USA) [14]. Genes were considered significantly differentially expressed when the q‐value was ≤ 0.05. Genes meeting this criterion and showing a log2 fold change (log2FC) ≤ −1 or ≥ 1 between FAM20A cells and control cells were selected for further analysis. The RNA sequencing data files are available at Gene Expression Omnibus (GEO accession: GSE289188).

### 2.5 Gene Ontology Analysis

Differentially expressed genes (DEG) were analysed using Fisher's exact test to determine significant enrichment in Gene Set Enrichment Analysis within The Database for Annotation, Visualisation, and Integrated Discovery v6.7 (DAVID, http://david.abcc.ncifcrf.gov). Gene Ontology (GO) terms encompassing biological processes, cellular components, and molecular functions, as well as Kyoto Encyclopedia of Genes and Genomes (KEGG) pathways and Reactome pathways, were considered significant with a *p‐*value < 0.05.

### 2.6 Protein–Protein Interaction Analysis

The DEG of biological processes was further examined using the Search Tool for the Retrieval of Interacting Genes database (STRING) (https://string‐db.org/) to demonstrate the protein–protein interaction networks. Within the STRING network, nodes represent genes and edges connecting these nodes represent predicted or experimentally determined PPIs. The colour of each edge signifies the specific evidence supporting the interaction: red: fusion events observed in cancer (potentially indicating functional dependency), green: genomic co‐localization (suggesting potential co‐regulation), blue: co‐expression across various datasets (implying involvement in similar biological processes), purple: experimentally validated protein–protein interactions (PPIs) (providing the most robust evidence), yellow: interactions curated from scientific literature (supporting potential functional connections), light blue: interactions documented in public PPI databases (providing additional evidence), and black: co‐expression patterns observed in expression studies (suggesting co‐regulation or shared pathways).

### 2.7 Quantitative Real‐Time Polymerase Chain Reaction (qRT‐PCR)

To validate the RNA sequencing data, qRT‐PCR was conducted. RNA samples were reverse transcribed to cDNA using iScript Reverse Transcription Supermix (Bio‐rad, CA, USA). The mRNA expression levels were quantified using the SYBR Green detection system (FastStart Essential DNA Green Master; Roche Diagnostic, CA, USA). β‐actin served as the endogenous control. Expression levels were normalised to the endogenous control and calculated using the 2^‐∆∆Cq^ method. The primer sequences used in this study, including *FAM20A* and genes related to biological processes identified in RNA Sequencing analysis, are shown in Supplementary Table S1.

### 2.8 Cell Adhesion and Spreading

Cells were seeded on glass coverslips and cultured for 30 min, 2 h, 6 h, or 24 h. Following the respective incubation periods, the cells were gently washed with phosphate‐buffered saline (PBS) and fixed with 3% glutaraldehyde (Sigma‐Aldrich, MA, USA) for 30 min at room temperature. Subsequently, the cells were dehydrated in a graded series of ethanol, followed by treatment with hexamethyldisilazane (HMDS), sputter‐coated with gold and examined using a Scanning Electron Microscope (Quanta 250, FEI, Hillsboro, OR, USA). The criteria for categorising cell spreading were applied as previously described [15, 16]. Briefly, morphological progression was classified into four distinct stages. Stage 1 was defined as round cells with or without a few filopodia, indicating early adhesion. Stage 2 included cells with numerous cytoplasmic extensions, signifying initial spreading. Stage 3 was characterised by circumferential extension of lamellipodia with a dome‐shaped central morphology, representing intermediate spreading. Stage 4 comprised fully flattened and spread cells, reflecting stable attachment. This classification allowed for the assessment of cellular dynamics and identification of delayed behaviours in attachment and spreading processes.

### 2.9 Cell Differentiation

The cells were cultured in osteogenic medium, which consisted of growth medium supplemented with 50 μg/mL ascorbic acid, 10 mM beta‐glycerophosphate and 100 nM dexamethasone for a duration of 21 days. Cells cultured in growth medium (GM) were used as a control. The background osteogenic markers were measured (SI Table S7). The mineral deposition (Day 21) and mRNA expression levels of *FAM20A* and *ALP*, early osteogenic differentiation markers, on Day 7 were examined.

### 2.10 Alizarin Red S Staining

Following 21 days of osteogenic induction, the cells were fixed with cold methanol for 10 min and subsequently washed with deionised water. The cells were then stained with a 2% solution of alizarin red S (Sigma, MA, USA) at room temperature, washed again with deionised water and allowed to air‐dry. Staining intensity was quantified using 10% cetylpyridium chloride in 10 mM sodium phosphate, and the absorbance of the resulting product was measured using a microplate reader at 570 nm.

### 2.11 MTT Assay

Cells were seeded at a density of 5 × 10^3^ cells per well in 48‐well plates. After 3, 7, or 10 days, 0.5 mg/mL 3‐(4,5‐dimethylthiazol‐2‐yl)‐2,5‐diphenyltetrazolium bromide (MTT) (Merck, Darmstadt, Germany) was added to the cells. The plates were then incubated for 1 h at 37°C. After incubation, the formazan crystals formed were dissolved in dimethyl sulfoxide (DMSO), and the level of formazan was measured at 570 nm using a microplate reader.

### 2.12 Colony Forming Assay

Single‐cell suspensions were seeded in 6‐well plates at a density of 500 cells per well and cultured in DMEM with 10% FBS at 37°C with 5% CO_2_. After 14 days, the cells were fixed with 100% methanol for 10 min and stained with 1% crystal violet.

### 2.13 Cell Cycle

Cells were seeded at 3 × 10^5^ cells/well and allowed to attach and grow for 24 h under standard culture conditions. At the time of analysis, both the attached and floating cells were harvested. The cells were fixed by gently adding 70% ice‐cold ethanol and stored at 4°C for 30 min. RNA was eliminated by adding 2 μL of 4 mg/mL RNase A (Thermo Fisher Scientific, MA, USA). The cells were then stained with 4 μg/mL propidium iodide and analysed using a FACSCalibur flow cytometer with CellQuest software (BD Bioscience, NJ, USA).

### 2.14 Cell Apoptosis

Cell apoptosis was determined using a FITC Annexin V apoptosis detection kit with propidium iodide (PI) (BioLegend, CA, USA) following the manufacturer's instructions. Confluent monolayer cells were harvested and washed with PBS. The cells were then resuspended in 100 μL of binding buffer containing 5 μL of Annexin V‐FITC and 10 μL of PI, followed by incubation for an additional 15 min at room temperature in the dark. Subsequently, 300 μL of binding buffer was added, and flow cytometric analysis was performed using a FACSCalibur flow cytometer (BD Bioscience, NJ, USA).

### 2.15 Flow Cytometry

Cells were stained for surface markers including CD44 (BioGenex, CA, USA), CD45 (BioGenex), CD90 (ImmunoTools, Friesoythe, Germany), CD105 (ImmunoTools) and CD73 (ImmunoTools) [17]. The expression levels of these markers were measured using a FACSCalibur flow cytometer (BD Biosciences, CA, USA).

### 2.16 Immunofluorescence Staining

Cells were seeded on chamber slides at a density of 5 × 10^3^ cells/well and cultured for 24 h. Following incubation, cells were washed and fixed with 4% ice‐cold paraformaldehyde for 10 min at room temperature, then rinsed three times with PBST (10 mM PBS containing 0.1% Triton X‐100; Sigma‐Aldrich, MO, USA) for 5 min each. To block non‐specific binding, cells were incubated with 1% BSA in PBS. Subsequently, cells were incubated with rhodamine–phalloidin (Invitrogen, CA, USA) diluted in blocking buffer. Fluorescence images were captured using a ZEISS fluorescence microscope (ZEISS, Oberkochen, Germany).

### 2.17 Statistical Analysis

The data are presented as the mean ± standard error of the mean (SEM) for each group, which included one patient and two control subjects. Three independent experiments were conducted for each condition (*n* = 3). To assess significant differences between the patient and control groups, we employed the one‐sided Mann–Whitney *U* test, with a significance threshold of *p* < 0.05. Statistical analyses were performed using GraphPad Prism 8 software (GraphPad Software, CA, USA).

## 3 Results

### 3.1 RNA Sequencing and Differential Gene Expression of Gingival Fibroblasts From an AI1G Patient

**FIGURE 1:**
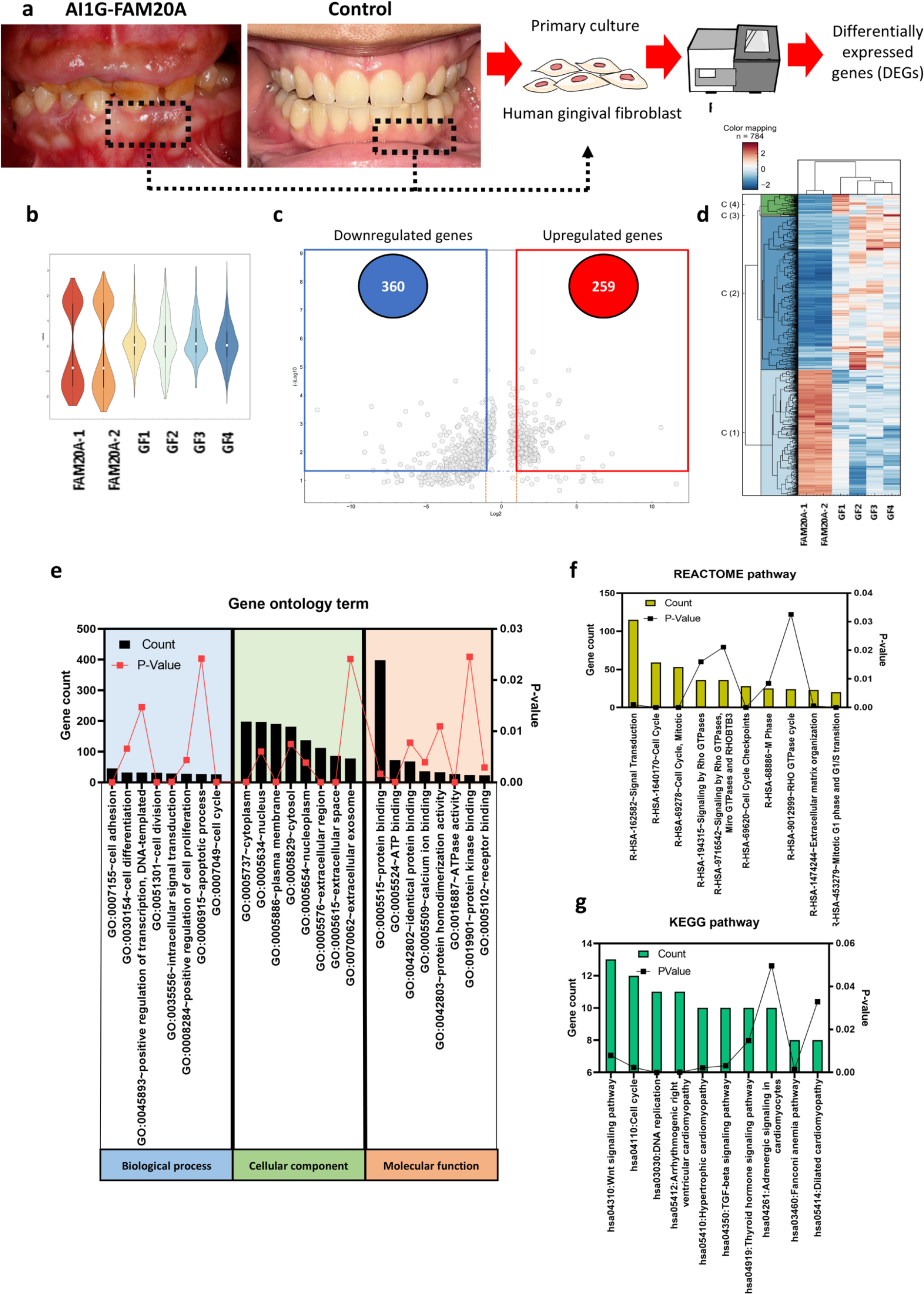
RNA‐sequencing profile of gingival fibroblast cells of AI1G (FAM20A cells) and control cells. (a) A schematic diagram illustrating the study design, comparing the transcriptomes of FAM20A and control cells. (b) Distribution of true significance levels for gene expression in each sample which shows the overall distribution of statistically significant genes. (c) Volcano plot of differentially expressed genes (DEGs) between FAM20A cells and controls. The abscissa shows the log2 (fold change) in gene expression, and the ordinate shows the −Log10(*q*‐value). Significantly upregulated genes are indicated in red boxes and downregulated genes are indicated in blue boxes, with a threshold of 1 ≥ Log2 (fold change) ≥ −1. (d) Heatmap showing the individual expression patterns of upregulated and downregulated genes. (e) Functional analysis of DEGs by Gene Ontology classifications. The top 8 significantly enriched GO terms are shown, categorised by biological process, cellular component, and molecular function. Analysis of DEGs using REACTOME (f) and KEGG (g) pathway databases. This identifies enriched pathways potentially affected by FAM20A mutations.

A patient with compound heterozygous variants c.1231C>T and c.1109+3_1109+7delinsTGGTC in *FAM20A* exhibited both AI and gingival overgrowth (Figure 1a). As previously demonstrated in our studies [6, 11], the patient's gingival fibroblasts, referred to as ‘FAM20A cells’, showed significantly reduced FAM20A mRNA and protein levels. To further investigate gingival pathophysiology, RNA sequencing was performed on gingival fibroblast cells obtained from the patient compared with four controls. Both FAM20A and control cells expressed MSC‐specific cell surface markers CD73, CD90 and CD44, but not the MSC‐negative marker CD45, in all cell samples analysed (SI Figure S1). The proportion and distribution of significantly expressed genes in each sample were determined, revealing two distinct groups of gene distributions between FAM20A and control cells (Figure 1b). Differentially expressed genes (DEGs) were identified between FAM20A and control cells using a significance threshold of *p* < 0.05 and fold changes > 1 or < −1. DEGs showed that 259 genes were upregulated, while 360 genes were downregulated in FAM20A cells compared with controls (Figure 1c,d).

### 3.2 Gene Ontology and Pathway Enrichment Analysis Revealed Roles of FAM20A in Gingival Fibroblast Function

To elucidate the functional roles of DEGs in FAM20A cells, Gene Ontology (GO) enrichment analysis was performed. This analysis revealed significant enrichment of DEGs in specific biological processes, cellular components and molecular functions (Figure 1e). Enriched biological processes included cell adhesion, differentiation, positive regulation of transcription, division, intracellular signal transduction, positive regulation of cell proliferation, apoptosis and cell cycle regulation. Cellular components significantly represented among the DEGs included the cytoplasm, nucleus, plasma membrane, cytosol, nucleoplasm, extracellular region, extracellular space and extracellular exosomes. Furthermore, enriched molecular functions included protein binding, ATP binding, calcium ion binding, protein homodimerisation activity, ATPase activity, protein kinase binding and receptor binding. These findings suggest that FAM20A plays a crucial role in regulating fundamental cellular processes and functions in gingival fibroblasts.

Pathway analysis using the Reactome and KEGG databases was performed to identify the signalling pathways affected by the DEGs. Reactome analysis revealed significant enrichment in pathways related to signal transduction, cell cycle (including checkpoints, M phase and G1/S transition), Rho GTPase signalling (including Rho GTPase cycle) and extracellular matrix organisation (Figure 1f). KEGG pathway analysis highlighted enrichment in pathways such as Wnt signalling, cell cycle, DNA replication, TGF‐beta signalling, thyroid hormone signalling and several pathways related to cardiomyopathies (arrhythmogenic right ventricular cardiomyopathy, hypertrophic cardiomyopathy, dilated cardiomyopathy), adrenergic signalling in cardiomyocytes and Fanconi anaemia (Figure 1g). In this study, we investigated deeper into the biological processes influenced by FAM20A to elucidate its specific roles within the enriched signalling pathways.

### 3.3 Delayed Initial Gingival Fibroblast Attachment and Spreading

**FIGURE 2:**
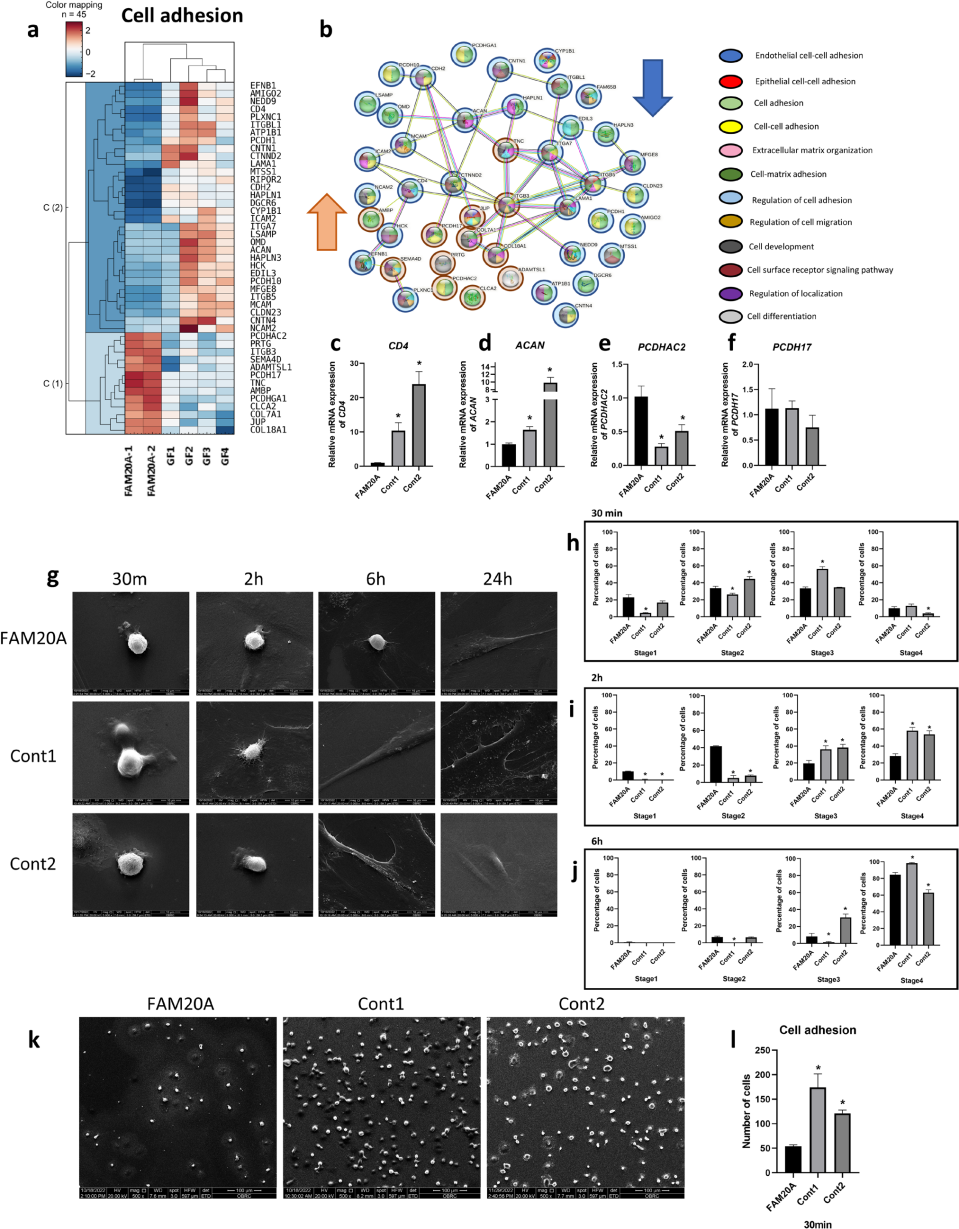
Impaired cell adhesion in FAM20A cells. (a) Heatmap illustrating the upregulated and downregulated genes involved cell adhesion in FAM20A and control cells. (b) Protein–protein interactions and associated biological processes among genes related to cell adhesion. (c–f) Confirmation of RNA sequencing results using real‐time PCR for two upregulated and two downregulated genes involved in cell adhesion. (g–j) Cell attachment and spreading assay demonstrating delayed cell attachment in FAM20A cells compared to control cells; (g) Representative SEM images (i–j) Quantification of attached cells. (k, l) Cell attachment at 30 min: quantification of attached cells at the 30‐min time point reveals a significantly lower number of attached FAM20A cells compared to control cells.

The cell adhesion process was significantly enriched in the biological processes revealed by GO analysis. Of the 45 differentially expressed genes associated with these processes in FAM20A cells compared to controls, 32 were downregulated and 13 were upregulated (Figure 2a, SI Table S2). These genes are involved in various aspects of cell adhesion, including epithelial and endothelial cell–cell adhesion, cell–matrix adhesion, extracellular matrix organisation, cell development, cell surface receptor signalling and cell differentiation. Protein–protein interaction analysis using the STRING database revealed that 15 of the 45 corresponding proteins lacked interactions with other proteins within the dataset. ITGB3 exhibited the highest degree of connectivity, directly interacting with 11 other proteins, including CTNND2, ICAM2, CD4, COL7A1, COL18A1, LAMA1, ITGB5, MFGE8, ITGA7 and TNC (Figure 2b). To validate the RNA‐sequencing results, qRT‐PCR was performed on two of the most upregulated and two of the most downregulated genes. Consistent with the RNA‐Seq data, *CD4* and *ACAN* expressions were significantly lower in FAM20A cells compared to controls (Figure 2c,d), while *PCDHAC2* expression was significantly higher (Figure 2e). However, *PCDH17* expression showed no significant difference (Figure 2f). Time‐lapse microscopy was used to assess further cell attachment and spread dynamics to investigate the impact of FAM20A insufficiency on cell adhesion. At 30 min, the majority of FAM20A cells were in stages 1 and 2, characterised by initial attachment and numerous cytoplasmic extensions. In contrast, control cells were predominantly in stages 2 and 3, exhibiting circumferential lamellipodia extension and a dome‐like morphology (Figure 2g,h). By 2 h, FAM20A cells remained predominantly in stage 2 (42%), while a significantly greater proportion of control cells had progressed to stage 4 (> 50%), indicating more advanced spreading and attachment (Figure 2g,i). By 6 h, the distribution of FAM20A cells across stages was similar to that of control cells, with the majority in stage 4 (Figure 2g,j). Furthermore, significantly fewer FAM20A cells were attached at 30 min than control cells (Figure 2k,l). These results suggest that FAM20A insufficiency delays gingival fibroblasts' initial attachment and spreading. Still, this delay is overcome over time, with cells eventually achieving a similar degree of spreading as control cells.

### 3.4 Impaired Osteogenic Differentiation in Gingival Fibroblasts

**FIGURE 3:**
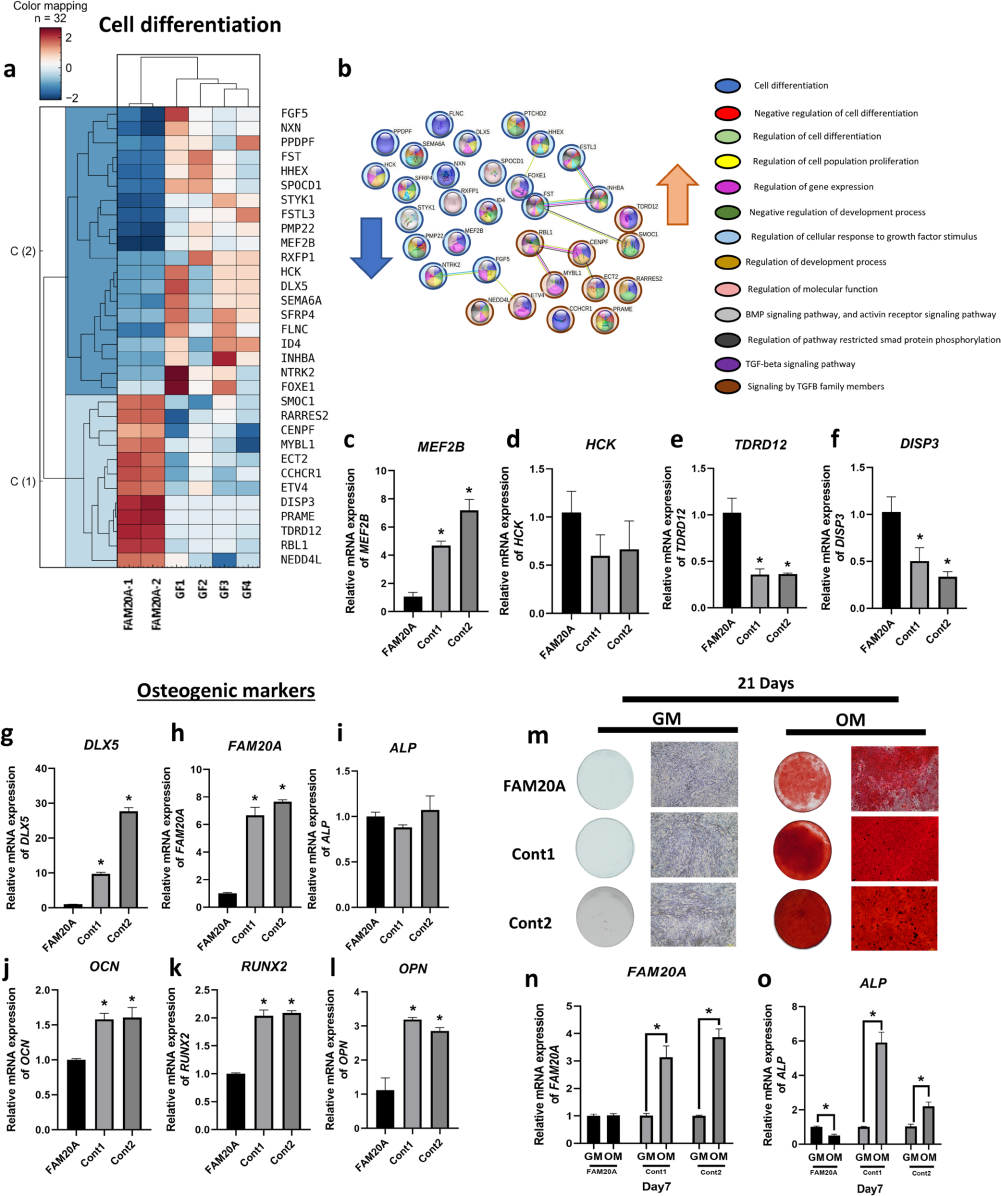
Disrupted cell differentiation in FAM20A cells. (a) Heatmap showing the upregulated and downregulated genes involved in cell differentiation in FAM20A and control cells. (b) Protein–protein interactions and associated biological processes among genes related to cell differentiation. (c–f) Confirmation of RNA sequencing results using real‐time PCR for two upregulated and two downregulated genes involved in cell differentiation. (g–l) Reduced mRNA expression levels of genes related to osteogenic differentiation (g) *DLX5*, (h) *FAM20A*, (j) *OCN*, (k) *RUNX2*, and (l) *OPN* in FAM20A gingival fibroblast cells, with no significant difference observed for (i) *ALP* mRNA levels. (m) Alizarin S red staining of FAM20A and control cells after induction with osteogenic medium (OM) for 21 days. FAM20A cells exhibited decreased mineralization compared to control cells. (n) *FAM20A* mRNA expression in FAM20A cells did not show a significant difference, whereas both control cell lines displayed upregulation after induction with OM for 7 days. (o) *ALP* mRNA expression in FAM20A cells showed a significant decrease, while both control cells revealed upregulation after induction with OM for 7 days.

Cell differentiation was the second most enriched biological process. Of the 32 differentially expressed genes associated with this process in FAM20A cells compared to controls, 12 were upregulated and 20 were downregulated (Figure 3a, SI Table S3). Protein–protein interaction analysis indicated that 18 of the 45 corresponding proteins lacked interactions within the dataset (Figure 3b). Proteins such as INHBA, FST, RBL1, CENPF and FGF5 each interacted with two other proteins, suggesting their involvement in cell differentiation regulatory networks. To validate the RNA‐Seq data, qRT‐PCR was performed on two of the most upregulated and two of the most downregulated genes. Consistent with the RNA‐Seq data, *MEF2B* expression was significantly lower in FAM20A cells compared to controls (Figure 3c), and *TDRD12* and *DISP3* expression was significantly higher (Figure 3e,f). However, *HCK* expression showed no significant difference between the two groups, despite the downregulation observed in the RNA‐Seq data (Figure 3d). RNA‐Seq analysis also revealed significant downregulation of *DLX5*, a key regulator of osteogenic differentiation. To further investigate this, basal mRNA expression levels of FAM20A and osteogenic markers (*DLX5*, *OCN*, *RUNX2* and *OPN*) were assessed in FAM20A and control cells (Figure 3g,h,j–l). These markers were all significantly downregulated in FAM20A cells, suggesting impaired osteogenic differentiation potential. *ALP* expression, however, showed no significant difference between the two groups (Figure 3i).

Our comprehensive research on FAM20A and control cells cultured in osteogenic medium (OM) for 21 days has unveiled significant findings. Alizarin Red S staining revealed reduced mineral deposition in the FAM20A cells compared to controls, indicating impaired mineralisation capacity (Figure 3m). Furthermore, after 7 days of OM induction, *ALP* mRNA expression, an early osteogenic differentiation marker, was significantly upregulated in control cells compared to cells cultured in growth medium (GM). In contrast, *ALP* expression was significantly lower in OM‐induced FAM20A cells compared to FAM20A cells in GM, while FAM20A expression itself showed no significant change in response to OM (Figure 3n,o). These findings further support the hypothesis that FAM20A insufficiency may impair osteogenic differentiation in gingival fibroblasts. The inability of these cells to express sufficient FAM20A appears to compromise their osteogenic potential, providing insights into the molecular mechanisms underlying this process.

### 3.5 Enhanced Cell Cycle Progression and Proliferation in Gingival Fibroblasts

**FIGURE 4:**
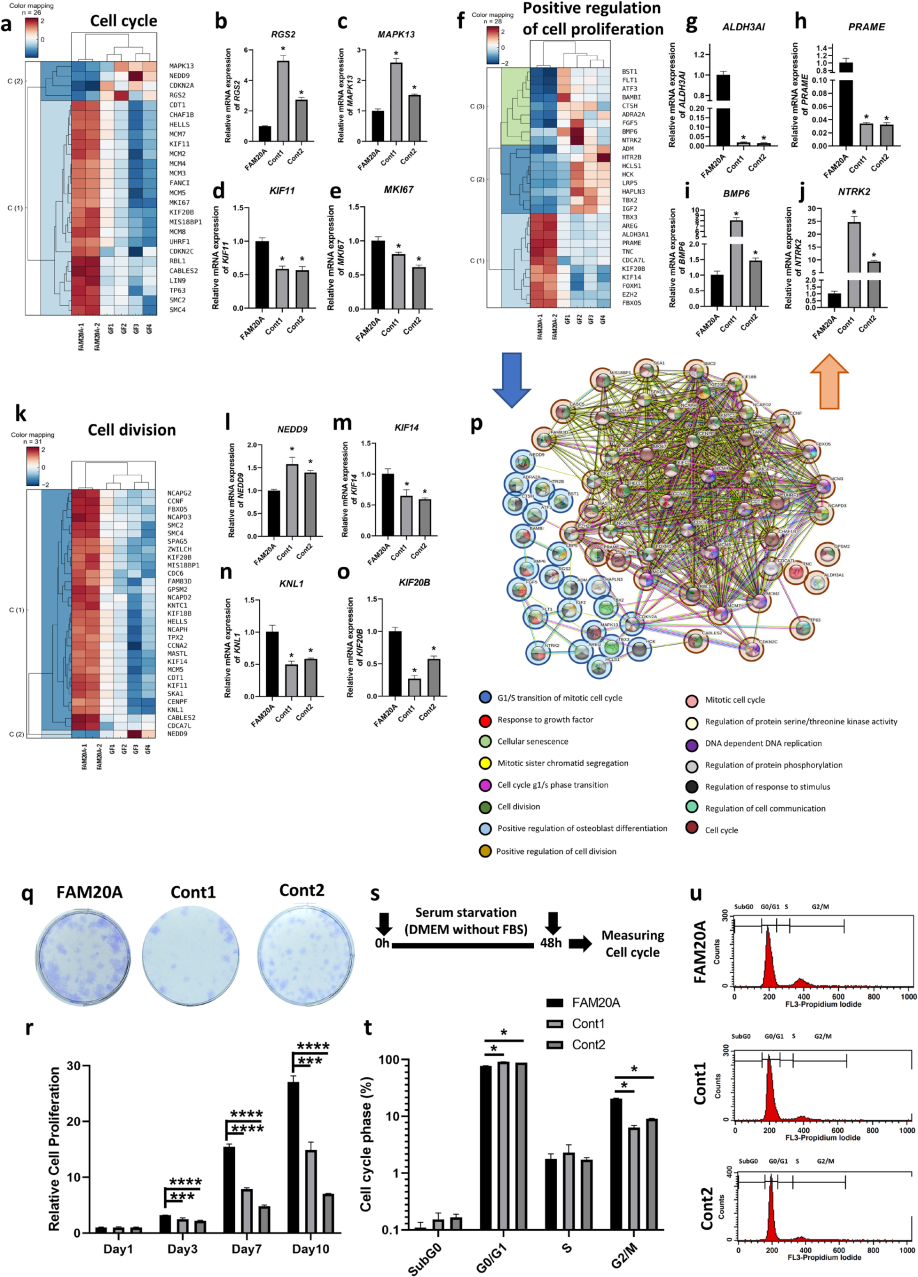
Dysregulation of cell proliferation, cell division, and cell cycle in FAM20A cells. (a) Heatmap illustrates the upregulated and downregulated genes involved in cell cycle in FAM20A and control cells. (b–e) Confirmation of RNA sequencing results using real‐time PCR for two upregulated and two downregulated genes involved in cell cycle. (f) Heatmap showing the upregulated and downregulated genes involved in positive regulation of cell proliferation in FAM20A and control cells. (g–j) Confirmation of RNA sequencing results using real‐time PCR for two upregulated and two downregulated genes involved in positive regulation of cell proliferation. (k) Heatmap showing the upregulated and downregulated genes involved in cell division in FAM20A and control cells. (l–o) Confirmation of RNA sequencing results using real‐time PCR for genes involved in cell division. (p) Protein–protein interactions and associated biological processes among genes related to positive regulation of cell proliferation, cell cycle, and cell division. (q) FAM20A cells exhibited a higher capacity to form colonies compared with control cells. (r) MTT assay was performed to measure cell proliferation. FAM20A cells showed significantly increased proliferation on Days 3, 7, and 10 compared to controls. (s) Schematic diagram illustrating the cell cycle experiment design. (t, u) Cell cycle analysis revealed a significantly higher proportion of FAM20A cells in the G2/M phase compared to controls, while a significantly lower proportion was observed in the G0/G1 phases. This suggests a more active cell cycle in FAM20A cells.

GO analysis of biological processes revealed the enrichment of interconnected pathways related to cell proliferation, including positive regulation of cell proliferation, cell cycle, and cell division, with overlapping genes observed among these categories (Figure 1e, SI Tables S3–S5, SI Figure S2). Within the cell cycle processes, 22 genes were upregulated and 4 were downregulated in FAM20A cells compared to controls (Figure 4a, SI Table S4). The validation of these RNA‐Seq findings through qRT‐PCR provides a reassuring confirmation, demonstrating significantly increased expression of *KIF11* and *MKI67* (two of the most upregulated genes) and significantly decreased expression of *RGS2* and *MAPK13* (two of the most downregulated genes) in FAM20A cells (Figure 4b–e).

Our meticulous analysis of genes associated with positive regulation of cell proliferation revealed 11 upregulated and 17 downregulated genes in FAM20A cells compared to controls (Figure 4f, SI Table S5). The accuracy of our findings is further validated by the confirmation of our RNA‐Seq results through qRT‐PCR. We observed a significant increase in the expression of *ALDH3A1* and *PRAME*, two of the most upregulated genes, and significantly decreased expression of *BMP6* and *NTRK2*, two of the most downregulated genes (Figure 4g–j).

For cell division processes, 30 genes were upregulated and 1 was downregulated in FAM20A cells (Figure 4k, SI Table S6). qRT‐PCR validated the upregulation of *KIF14* and *KNL1* and the downregulation of *NEDD9* (Figure 4l–n). *KIF20B*, involved in all three GO categories (cell proliferation, cell cycle and cell division), was also significantly upregulated (Figure 4o, SI Figure S2). Our comprehensive protein–protein interaction analysis across these three processes, which included most proteins, with the exception of ADRA2A, CTSH, HTR2B, BST1, TGS2, TNC, GPSM2 and ALDH3A1, provided a thorough understanding of these processes (Figure 4p).

Our investigation into the functional consequences of gene expression changes involved a series of rigorous experiments. Cell proliferation, colony formation, and cell cycle analyses were performed. The results were striking. FAM20A cells formed significantly larger colonies than controls, indicating increased cell division capacity (Figure 4q). Cell proliferation assays revealed substantially higher proliferation rates in FAM20A cells at days 3, 7 and 10 (Figure 4r). Following 48 h of serum starvation, cell cycle analysis demonstrated a significant decrease in the G0/G1 population and a significant increase in the G2/M population in FAM20A cells compared to controls, suggesting reduced quiescence and enhanced cell cycle progression (Figure 4s–u). These findings underscore the pivotal role of FAM20A in cellular proliferation and division processes.

### 3.6 Suppressed Apoptosis in Gingival Fibroblasts

**FIGURE 5:**
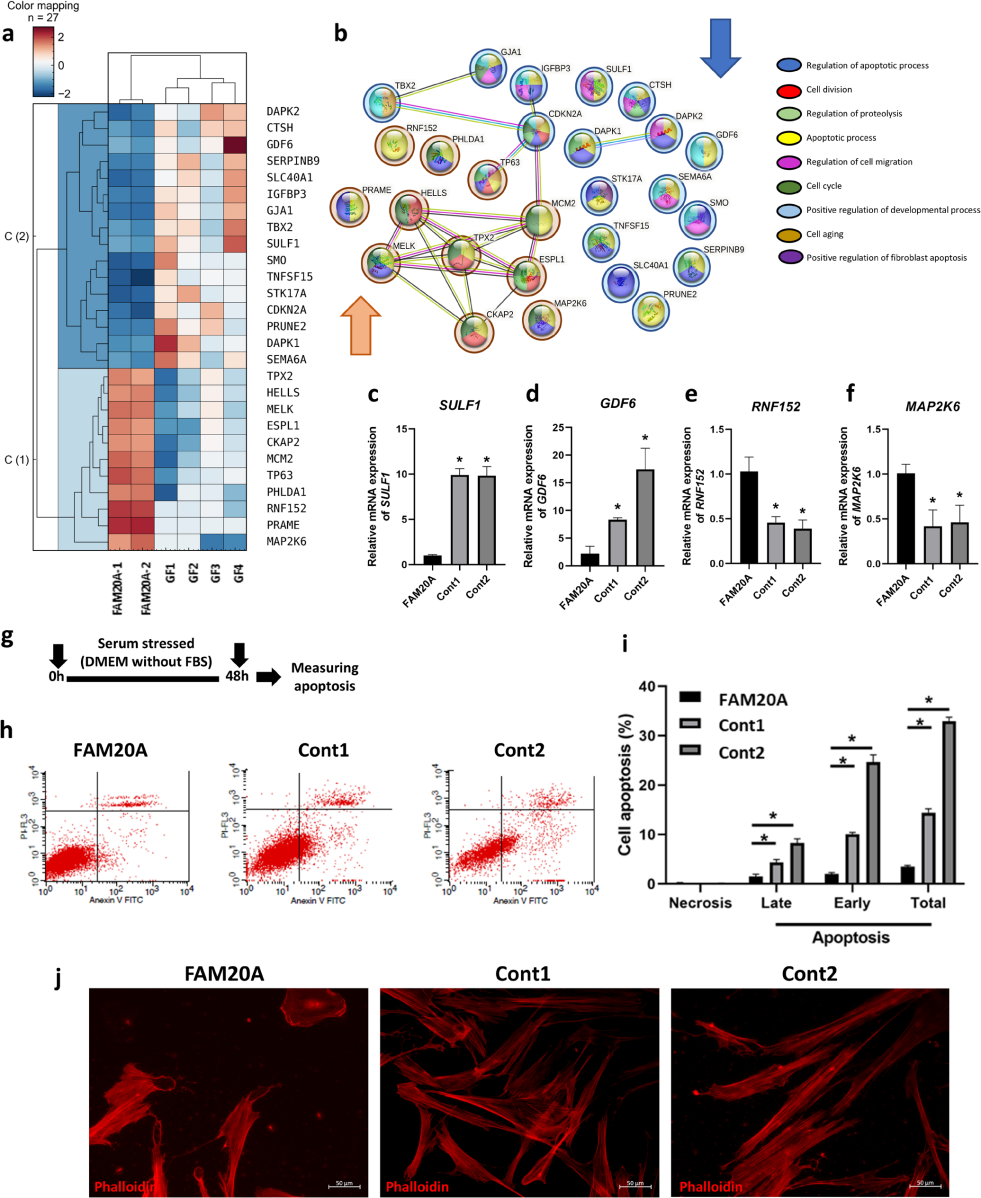
Reduced apoptosis in FAM20A cells. (a) Heatmap illustrates the upregulated and downregulated genes involved in cell apoptosis in FAM20A and control cells. (b) Protein–protein interactions and associated biological processes among genes related to cell apoptosis. (c–f) Confirmation of RNA sequencing results using real‐time PCR for two upregulated and two downregulated genes involved in cell apoptosis. (g) Schematic diagram illustrating the experimental design for analysing cell apoptosis. (h, i) FAM20A cells exhibited significantly lower levels of cell apoptosis, including late, early, and total apoptosis, compared to control cells. (j) Immunofluorescence staining revealed disorganised Actin cytoskeleton in FAM20A‐deficient cells compared to controls.

Our research has rigorously examined the role of apoptosis in FAM20A cells, uncovering its impact on biological processes. We found that 11 genes associated with apoptosis were upregulated and 16 were downregulated compared to controls (Figure 5a, Supplementary Table S7). Our protein–protein interaction analysis, conducted with meticulous care, highlighted TPX2, HELLS, MELK, ESPL1 and MCM2 as highly interactive proteins (Figure 5b). Fourteen of the apoptosis‐related proteins lacked detectable interactions within the dataset, suggesting they may function independently or through interactions not captured in this analysis. The validation of our findings through qRT‐PCR further confirms the thoroughness of our research, demonstrating significantly decreased expression of *SULF1* and *GDF6* (two of the most downregulated genes) and significantly increased expression of *RNF152* and *MAP2K6* (two of the most upregulated genes) in FAM20A cells (Figure 5c–f).

To further investigate the role of FAM20A in apoptosis, an Annexin V/PI apoptosis assay was performed. Following 48 h of serum starvation, FAM20A cells exhibited significantly lower levels of early, late and total apoptosis compared to control cells. Importantly, there was no significant difference in necrosis levels (Figure 5h,i). These results suggest that FAM20A may hold the key to protecting against serum starvation‐induced apoptosis.

### 3.7 Alteration of Extracellular Matrix Pattern

Immunofluorescence staining revealed that the cells exhibited reduced attachment and spreading on the culture surface. Notably, there were distinct differences in actin cytoskeleton organisation between the two groups. Control cells displayed well‐organised, prominent stress fibres, indicative of intact cytoskeletal structure and proper ECM engagement. In contrast, FAM20A cells showed loose, disorganised, or diminished actin filament structures. These phenotypic changes suggest alterations in ECM–cell interactions, consistent with the enriched DEGs in REACTOME and are often linked to alterations in Rho GTPase signalling, a key regulator of actin dynamics and cell adhesion [18, 19]. These alterations may reflect compromised ECM organisation and impaired cell‐matrix interactions associated with FAM20A insufficiency.

## 4 Discussion

The pathophysiology of gingival fibromatosis, a hallmark of AI1G associated with *FAM20A* variants, remains poorly understood. This study proposes the first evidence of altered cellular behaviour and molecular expression in AI1G gingival fibroblasts with FAM20A insufficiency. The discovery of significant differential expression of genes and proteins involved in cell adhesion, spreading, proliferation, cell cycle regulation, apoptosis and osteogenic differentiation, as revealed by RNA sequencing, is a significant breakthrough. These findings were further validated by in vitro assays, adding another layer of novelty to our research. These results suggest a complex interplay between these cellular processes in AI1G gingival fibroblasts with *FAM20A* insufficiency, potentially contributing to the gingival fibromatosis phenotype.

FAM20A insufficiency is associated with a significant alteration in the gingival fibroblast transcriptome. Differential expressions of genes and proteins involved in integrin signalling (e.g., *ITGA7, ITGB3, ITGB5, ITGBL1*), cadherin‐mediated adhesion (e.g., *CDH2, PCDH1, PCDH10*), ECM–receptor interactions (e.g., fibronectin, laminin, collagen) and focal adhesion pathways were observed. These pathways are critical for ECM organisation, cytoskeletal regulation and integrin‐mediated cell adhesion. Disrupted cell–ECM interactions, with upregulation of ECM components like fibronectin and collagen, potentially lead to excessive ECM deposition and fibrosis, a hallmark of gingival enlargement [20, 21]. Altered integrin expression suggests dysregulation of integrin signalling, impacting cellular responses to the ECM, including adhesion, migration and gene expression [22]. Furthermore, dysregulation of cell adhesion molecules (CAMs) like MCAM, ICAM2 and NCAM2 may contribute to chronic inflammation and fibrosis [23]. Additionally, alterations in genes related to actin filament organisation suggest cytoskeletal dysregulation, which could enhance fibroblast activation and ECM production [24]. These disruptions in integrin, cadherin and cytoskeletal regulation may impair cell adhesion and migration, promote aberrant proliferation and ultimately disrupt tissue architecture [25, 26].

The changes in gene expression patterns within FAM20A cells revealed the induction of genes involved in cell proliferation, cell division and cell cycle progression, along with a reduction in cell apoptosis. Our study identified increased expression of minichromosome maintenance proteins (MCMs), which are essential for DNA replication initiation and elongation, in FAM20A cells. Notably, the upregulation of *MCM2* and *MCM5* aligns with findings from previous studies in gingival fibromatosis patients [27]. Additionally, high expression levels of *MCM2*, *MCM3* and *Ki‐67* have been reported in ameloblastoma [28]. Several kinesins involved in mitosis or intracellular transport were also upregulated in FAM20A cells, suggesting their potential role in cell division and proliferation [29, 30]. These findings suggest that changes in cell cycle dynamics, including proliferation, division, and apoptosis, may be involved in the development of gingival fibromatosis.

The cytoskeletal alterations in FAM20A‐deficient cells, particularly the disorganisation of actin stress fibres, support a model of disrupted cell‐ECM communication and impaired cellular function [18, 31]. Consistent with this, transcriptomic analysis of ERS patients has revealed significant alterations in ECM organisation, angiogenesis and biomineralisation, which aligns with the dense collagen accumulation observed in connective tissues. These findings suggest FAM20A plays a key regulatory role in ECM homeostasis by balancing collagen production and degradation [10]. Previous reports have described characteristic histological features in AI1G/ERS gingiva, including disorganised collagen fibre bundles, myofibroblasts, remnants of odontogenic epithelium and psammomatous calcifications [10, 32]. Furthermore, in vitro culture of gingival fibroblasts from AI1G/ERS patients confirms these histopathological observations, demonstrating cellular programmes that contribute to aberrant calcification and altered extracellular matrix homeostasis [12].

The actin cytoskeleton is critical for cell shape, adhesion and motility, with its integrity tightly regulated by signals from integrins and the ECM [18, 33]. Building on this, the immunofluorescence study revealed that FAM20A‐deficient fibroblasts display reduced spreading and a disorganised actin network. These changes indicate cytoskeletal instability, consistent with impaired integrin‐mediated adhesion and spreading. Such instability can influence nuclear signalling and gene expression, which aligns with our transcriptomic data showing the upregulation of cell cycle and mitotic regulators. Collectively, these data suggest that FAM20A deficiency disrupts both ECM organisation and cytoskeletal dynamics, leading to defects in cell adhesion, proliferation and tissue remodelling.

Moreover, the altered expression of multiple genes (*INHBA*, *FSTL3*, *FST*, *ID4*, *RBL1* and *NEDD4L*) within the TGFβ pathway was observed. TGFβ signalling can have both positive and negative effects on bone and enamel formation, as well as promote tissue fibrosis, depending on the cellular context and the specific genes involved [34, 35, 36]. Proteomics analysis of conditioned media from ERS gingival fibroblasts revealed the induction of *TGFβ2* expression [9]. This upregulation of *TGFβ2* further increased the expression of downstream targets in the TGFβ signalling pathway, including collagens, matrix metallopeptidase 2 and fibronectin. Consequently, this could lead to alterations in both mineralisation and fibrosis [9]. Overexpression of growth factors such as TGF‐β and cytokines can result in excessive collagen production and fibrosis in the gingival tissues [36].

Additionally, our findings suggest a potential disruption of osteogenic differentiation in AI1G. The downregulation of key osteogenic markers, including *OCN*, *RUNX2* and *OPN*, in FAM20A gingival fibroblasts indicates that FAM20A insufficiency might contribute to defects in osteogenesis [8]. Additionally, altered expression of genes associated with cell differentiation, particularly those involved in BMP and TGFβ signalling pathways, both critical for osteoblast and ameloblast differentiation, further supports this hypothesis [37, 38]. In our study, the observed downregulation of *SFRP4*, a WNT signalling inhibitor, in FAM20A gingival fibroblasts aligns with findings in Pyle disease, a condition characterised by delayed tooth eruption caused by biallelic *SFRP4* variants [39]. Similarly, AI1G patients with biallelic *FAM20A* variants also exhibit delayed or failed tooth eruption [5], suggesting a potential interplay between *SFRP4* and *FAM20A* in AI1G pathophysiology. Furthermore, our study revealed the downregulation of *DLX5* and *LRP6*, a key component of WNT signalling, alongside reduced mineralisation. These findings suggest an imbalance in osteogenesis and altered WNT signalling associated with FAM20A insufficiency, which may play a role in tooth eruption failure [40, 41, 42, 43].

Previous studies in knockout mouse models have demonstrated that FAM20A interacts with FAM20C to regulate the phosphorylation of secreted proteins critical for biomineralisation, highlighting its key function in ameloblasts [44, 45]. In deciduous dental pulp cells derived from an AI1G patient, FAM20A deficiency led to impaired cellular behaviours, including reduced attachment, spreading, migration and colony formation, alongside downregulation of osteogenic genes and dysregulated inflammatory responses [8]. Moreover, transcriptomic profiling of dental tissues from ERS patients revealed disruptions in BMP, SMAD and WNT signalling pathways [13], suggesting that FAM20A is essential for maintaining pulp tissue homeostasis and contributes broadly to connective tissue regulation.

**FIGURE 6:**
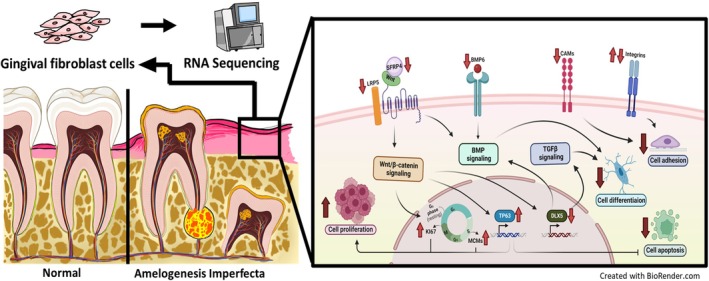
Integrated view: major molecular and cellular responses to FAM20A insufficient in gingival fibroblast cells.

Together with our current findings in gingival fibroblasts, which show aberrant ECM‐receptor interactions, altered focal adhesion signalling and diminished osteogenic potential, it becomes evident that FAM20A governs shared regulatory pathways across different dental cell types. These pathways, particularly WNT, BMP and TGFβ, are crucial for tissue remodelling and mineralisation [46]. Thus, FAM20A deficiency may drive both gingival fibromatosis and defective tooth eruption in AI1G through a common mechanism involving disrupted cell‐matrix interactions and signalling imbalances (Figure 6).

This study's limitation is that we included only one AI1G patient due to the difficulty in obtaining gingival fibroblast cells from patients with this condition. The urgency for future studies involving a larger cohort is underscored, as it would provide a more comprehensive understanding of the cellular and molecular mechanisms underlying the gingival pathologies and failure of tooth eruption associated with FAM20A insufficiency.

In conclusion, this study reveals a key role for FAM20A in regulating gingival fibroblast function, shedding light on how its insufficiency contributes to fibrotic remodelling and cellular dysfunction. These alterations likely underlie gingival overgrowth and may be associated with disrupted osteogenesis and tooth eruption failure in AI1G patients. Our findings enhance the mechanistic understanding of AI1G‐related gingival pathology and suggest potential molecular targets for future therapeutic strategies.

## Author Contributions

K.S. and T.P. contributed to conceptualisation, data analysis, prepared the original draft. S.T., C.K., P.S., S.P., N.S., S.P. performed the experiments, collected and analysed the data. V.S., H.S.J. provided critical comments on the manuscript. All authors critically revised and approved the manuscript.

## Ethics Statement

This study was approved by the Institutional Review Board, Faculty of Medicine, Chulalongkorn University (IRB 813/63) and in accordance with the 1964 Helsinki declaration and its later amendments. Written informed consent was obtained from all participants. Medical examinations and laboratory investigations were conducted.

## Conflicts of Interest

The authors declare no conflicts of interest.

## Supporting information


**Data S1.** Supporting Information.

## Data Availability

The RNA sequencing data are available in the Gene Expression Omnibus (GEO) under accession number GSE289188.
