# Supplementary material for: FAM20A Deficiency Drives Transcriptomic Dysregulation and Functional Impairment in Gingival Fibroblasts
Source: Cell Prolif. 2025 Jul 22;59(2):e70096. doi: 10.1111/cpr.70096 (PMC12877953; doi:10.1111/cpr.70096)
Supplement: Supplementary file 1 — Data S1. Supporting Information. [file CPR-59-e70096-s001.docx]

**Supplementary Data**

**FAM20A Deficiency Drives Transcriptomic Dysregulation and Functional Impairment in Gingival Fibroblasts**

**Supplementary Table S1.** **Primer sequences.**

| **Primers** |  | **Primer Sequences (5’->3’)** |
| --- | --- | --- |
| ***FAM20A*** | Forward | GGAACAGGCGACACAAGATG |
|  | Reverse | CTGATGGTGGGAAAGTGCCT |
| ***ALP*** | Forward | CGAGATACAAGCACTCCCACTTC |
|  | Reverse | CTGTTCAGCTCGTACTGCATGTC |
| ***RUNX2*** | Forward | ATGATGACACTGCCACCTCTGA |
|  | Reverse | ATGGAGGGCGGATTGGAAA |
| ***OPN*** | Forward | AGGAGGAGGCAGAGCACA |
|  | Reverse | CTGGTATGGCACAGGTGATG |
| ***OCN*** | Forward | CTTTGTGTCCAAGCAGGAGG |
|  | Reverse | CTGAAAGCCGATGTGGTCAG |
| ***CD4*** | Forward | CCAGAGGCCCTGCCATTTC |
|  | Reverse | GCCCAGCACCACTTTCTTTC |
| ***ACAN*** | Forward | CAAGGAGACAGAGGGACACG |
|  | Reverse | AGGTGGCTCCATTCAGACAAG |
| ***PCDHAC2*** | Forward | AGCGGAGGAATAAGAGAAGCAG |
|  | Reverse | AAGGTCCCCGTGTCTTTTG |
| ***PCDH17*** | Forward | ACTAATGCAAGCGAGACCCC |
|  | Reverse | TCTGGGTCCTTAAACGTGGAG |
| ***TDRD12*** | Forward | CGTTTTCAGTGCTTGGGTGG |
|  | Reverse | CTACCTTGGGGAAGTGGCTG |
| ***MEF2B*** | Forward | CGAGGGGGACTAAACACCTC |
|  | Reverse | CCAGGCCATATTCTGGGGG |
| ***DISP3*** | Forward | CAGCAGCTTTGACCTCTTCA |
|  | Reverse | GCAACATCTGCAGGAAGGA |
| ***HCK*** | Forward | GGAAGGGACTCAGACAGTGC |
|  | Reverse | ATGCACCCCATCCTGGGC |
| ***DLX5*** | Forward | CAACTTTGCCCGAGTCTTCA |
|  | Reverse | GTTGAGAGCTTTGCCATAGGAA |
| ***RGS2*** | Forward | AATTCTGGCTGGCCTGTGAA |
|  | Reverse | GCATGAGGCTCTGTGGTGAT |
| ***MAPK13*** | Forward | GCTGCCCAAGACCTACGTG |
|  | Reverse | TCACCACGTAGCCAGTCATC |
| ***KIF11*** | Forward | GATGGACGTAAGGCAGCTCA |
|  | Reverse | TGTGGTGTCGTACCTGTTGG |
| ***MKI67*** | Forward | CGTCCCAGTGGAAGAGTTGT |
|  | Reverse | CGACCCCGCTCCTTTTGATA |
| ***ALDH3A1*** | Forward | TTACCGGGAGAGGCTGTGT |
|  | Reverse | GAAGGGGTAGTTCCAGGTGC |
| ***PRAME*** | Forward | TGCTGATGAAGGGACAACAT |
|  | Reverse | CAGCACTTGAAGTTTCCACCT |
| ***BMP6*** | Forward | GTCTTACAGGAGCATCAGCACAG |
|  | Reverse | GGAGTCACAACCCACAGATTG |
| ***NTRK2*** | Forward | ACTGTGAAAGGCAACCCCAA |
|  | Reverse | AGACCGAGAGATGTTCCCGA |
| ***NEDD9*** | Forward | TGTGTTGCCTCTCAAACTCCTC |
|  | Reverse | GCCTGAGCAAGGAGCAGG |
| ***NCAPD2*** | Forward | TGCTTAATGCCTACCGCCAA |
|  | Reverse | CACTGAATGGTCCCAACCGA |
| ***KIF14*** | Forward | GTTGGCATTAAGAGTTTAGCAGATA |
|  | Reverse | TTAGGGGTAAGGGGCATGTCT |
| ***KNL1*** | Forward | TGGGGTGTCTTCAGAGGCTA |
|  | Reverse | CCTGTTTCTGTTTCTTCCATTTCT |
| ***KIF20B*** | Forward | CCGGGAAAGTAAACTGACTCAC |
|  | Reverse | TTCTAGCTCCTCAACCAAATCCT |
| ***SULF1*** | Forward | AATGAATCTTGGGGCCGGTG |
|  | Reverse | AGCAGAGATAAGGTGGCAGC |
| ***GDF6*** | Forward | GGCCGTCTTCCTCATCAGTT |
|  | Reverse | GCCGACTTGGAAGACTGGAA |
| ***RNF152*** | Forward | GCGCGTATCTAACCCTTCCT |
|  | Reverse | TAATGGCAAGCTCACAGGCA |
| ***MAP2K6*** | Forward | GCAGCACAGCCTTCCCTAA |
|  | Reverse | AGGGTTTCGCTTCTTGCCTT |
| ***β-actin*** | Forward | TGGAACGGTGAAGGTGACAG |
|  | Reverse | AACAACGCATCTCATATTTGGAA |

**Supplementary Table S2. Up and down-regulated genes in the cell adhesion process of FAM20A cells**

| **Gene name** | **Ensembl Gene ID** | **Description** | **Log2(fold-change)** |
| --- | --- | --- | --- |
| *ACAN* | ENSG00000157766 | aggrecan | -10.23 |
| *CD4* | ENSG00000010610 | CD4 molecule | -9.09 |
| *HCK* | ENSG00000101336 | HCK proto-oncogene, Src family tyrosine kinase | -7.59 |
| *HAPLN1* | ENSG00000140511 | hyaluronan and proteoglycan link protein 3 | -6.78 |
| *CNTN1* | ENSG00000144619 | contactin 1 | -6.25 |
| *RIPOR2* | ENSG00000111913 | RHO family interacting cell polarization regulator 2 | -4.91 |
| *CNTN4* | ENSG00000144619 | contactin 4 | -4.49 |
| *NCAM2* | ENSG00000154654 | neural cell adhesion molecule 2 | -4.05 |
| *CYP1B1* | ENSG00000138061 | cytochrome P450 family 1 subfamily B member 1 | -3.05 |
| *CTNND2* | ENSG00000169862 | catenin delta 2 | -2.97 |
| *PCDH1* | ENSG00000156453 | protocadherin 1 | -2.97 |
| *OMD* | ENSG00000127083 | osteomodulin | -2.87 |
| *LAMA1* | ENSG00000101680 | laminin subunit alpha 1 | -2.86 |
| *PCDH10* | ENSG00000138650 | protocadherin 10 | -2.77 |
| *MCAM* | ENSG00000076706 | melanoma cell adhesion molecule | -2.65 |
| *HAPLN3* | ENSG00000140511 | hyaluronan and proteoglycan link protein 3 | -2.61 |
| *ITGA7* | ENSG00000135424 | integrin subunit alpha 7 | -2.56 |
| *PLXNC1* | ENSG00000136040 | plexin C1 | -2.55 |
| *DGCR6* | ENSG00000183628 | DiGeorge Syndrome Critical Region Gene 6 | -2.37 |
| *ICAM2* | ENSG00000108622 | intercellular adhesion molecule 2 | -2.24 |
| *CDH2* | ENSG00000170558 | cadherin 2 | -2.23 |
| *EDIL3* | ENSG00000164176 | EGF like repeats and discoidin domains 3 | -1.96 |
| *LSAMP* | ENSG00000185565 | limbic system associated membrane protein | -1.88 |
| *NEDD9* | ENSG00000111859 | neural precursor cell expressed, developmentally down-regulated 9 | -1.86 |
| *CLDN23* | ENSG00000253958 | claudin 23 | -1.86 |
| *AMIGO2* | ENSG00000139211 | adhesion molecule with Ig like domain 2 | -1.74 |
| *MFGE8* | ENSG00000140545 | milk fat globule EGF and factor V/VIII domain containing | -1.61 |
| *ITGB5* | ENSG00000082781 | integrin subunit beta 5 | -1.46 |
| *MTSS1* | ENSG00000170873 | MTSS I-BAR domain containing 1 | -1.44 |
| *EFNB1* | ENSG00000090776 | ephrin B1 | -1.31 |
| *ITGBL1* | ENSG00000198542 | integrin subunit beta like 1 | -1.26 |
| *ATP1B1* | ENSG00000143153 | ATPase Na+/K+ transporting subunit beta 1 | -1.24 |
| *PCDHGA1* | ENSG00000204956 | protocadherin gamma subfamily A, 1 | 1.11 |
| *COL7A1* | ENSG00000114270 | collagen type VII alpha 1 chain | 1.14 |
| *SEMA4D* | ENSG00000187764 | semaphorin 4D | 1.15 |
| *JUP* | ENSG00000173801 | junction plakoglobin | 1.15 |
| *TNC* | ENSG00000041982 | tenascin C | 1.16 |
| *ADAMTSL1* | ENSG00000178031 | ADAMTS like 1 | 1.19 |
| *COL18A1* | ENSG00000182871 | collagen type XVIII alpha 1 chain | 1.3 |
| *CLCA2* | ENSG00000137975 | chloride channel accessory 2 | 1.37 |
| *ITGB3* | ENSG00000259207 | integrin subunit beta 3 | 1.59 |
| *AMBP* | ENSG00000106927 | alpha-1-microglobulin/bikunin precursor | 2.24 |
| *PRTG* | ENSG00000166450 | protogenin | 2.26 |
| *PCDHAC2* | ENSG00000243232 | protocadherin alpha subfamily C, 2 | 2.75 |
| *PCDH17* | ENSG00000118946 | protocadherin 17 | 5.39 |

**Supplementary Table S3. Up and down-regulated genes in the cell differentiation process of FAM20A cells**

| **Gene name** | **Ensembl Gene ID** | **Description** | **Log2(fold-change)** |
| --- | --- | --- | --- |
| *MEF2B* | ENSG00000213999 | myocyte enhancer factor 2B | -8.64 |
| *HCK* | ENSG00000101336 | HCK proto-oncogene, Src family tyrosine kinase | -7.59 |
| *NTRK2* | ENSG00000148053 | neurotrophic receptor tyrosine kinase 2 | -5.48 |
| *SFRP4* | ENSG00000106483 | secreted frizzled related protein 4 | -5.08 |
| *DLX5* | ENSG00000105880 | distal-less homeobox 5 | -4.84 |
| *FOXE1* | ENSG00000178919 | forkhead box E1 | -4.64 |
| *RXFP1* | ENSG00000171509 | relaxin family peptide receptor 1 | -3.88 |
| *ID4* | ENSG00000172201 | inhibitor of DNA binding 4, HLH protein | -3.72 |
| *PMP22* | ENSG00000109099 | peripheral myelin protein 22 | -2.46 |
| *HHEX* | ENSG00000152804 | hematopoietically expressed homeobox | -2.35 |
| *SEMA6A* | ENSG00000092421 | semaphorin 6A | -2.08 |
| *FSTL3* | ENSG00000070404 | follistatin like 3 | -2.05 |
| *STYK1* | ENSG00000060140 | serine/threonine/tyrosine kinase 1 | -1.78 |
| *INHBA* | ENSG00000122641 | inhibin subunit beta A | -1.63 |
| *FST* | ENSG00000134363 | follistatin | -1.53 |
| *FLNC* | ENSG00000128591 | filamin C | -1.32 |
| *FGF5* | ENSG00000138675 | fibroblast growth factor 5 | -1.25 |
| *NXN* | ENSG00000167693 | nucleoredoxin | -1.24 |
| *SPOCD1* | ENSG00000134668 | SPOC domain containing 1 | -1.04 |
| *PPDPF* | ENSG00000125534 | pancreatic progenitor cell differentiation and proliferation factor | -1.02 |
| *CCHCR1* | ENSG00000204536 | Coiled-Coil Alpha-Helical Rod Protein 1 | 1.01 |
| *ECT2* | ENSG00000114346 | epithelial cell transforming 2 | 1.04 |
| *ETV4* | ENSG00000175832 | ETS variant transcription factor 4 | 1.09 |
| *NEDD4L* | ENSG00000049759 | NEDD4 like E3 ubiquitin protein ligase | 1.14 |
| *RARRES2* | ENSG00000106538 | retinoic acid receptor responder 2 | 1.27 |
| *RBL1* | ENSG00000080839 | RB transcriptional corepressor like 1 | 1.27 |
| *MYBL1* | ENSG00000185697 | MYB proto-oncogene like 1 | 1.59 |
| *SMOC1* | ENSG00000198732 | SPARC related modular calcium binding 1 | 2.14 |
| *CENPF* | ENSG00000117724 | centromere protein F | 2.2 |
| *TDRD12* | ENSG00000173809 | tudor domain containing 12 | 4.47 |
| *DISP3* | ENSG00000204624 | dispatched RND transporter family member 3 | 6.74 |
| *PRAME* | ENSG00000185686 | PRAME Nuclear Receptor Transcriptional Regulator | 10.6 |

**Supplementary Table S4. Up and down-regulated genes in the cell cycle process of FAM20A cells**

| **Gene names** | **Ensembl Gene ID** | **Description** | **Log2(fold-change)** |
| --- | --- | --- | --- |
| *MKI67* | ENSG00000148773 | marker of proliferation Ki-67 | 2.46 |
| *KIF11* | ENSG00000138160 | kinesin family member 11 | 2.06 |
| *KIF20B* | ENSG00000138182 | kinesin family member 20B | 1.91 |
| *TP63* | ENSG00000073282 | tumor protein p63 | 1.82 |
| *FANCI* | ENSG00000140525 | FA complementation group I | 1.61 |
| *MCM8* | ENSG00000125885 | minichromosome maintenance 8 homologous recombination repair factor | 1.58 |
| *HELLS* | ENSG00000119969 | helicase, lymphoid specific | 1.43 |
| *MCM4* | ENSG00000104738 | minichromosome maintenance complex component 4 | 1.41 |
| *UHRF1* | ENSG00000276043 | ubiquitin like with PHD and ring finger domains 1 | 1.37 |
| *MCM2* | ENSG00000073111 | minichromosome maintenance complex component 2 | 1.37 |
| *LIN9* | ENSG00000183814 | lin-9 DREAM MuvB core complex component | 1.36 |
| *CDT1* | ENSG00000167513 | chromatin licensing and DNA replication factor 1 | 1.35 |
| *SMC4* | ENSG00000113810 | structural maintenance of chromosomes 4 | 1.33 |
| *RBL1* | ENSG00000080839 | RB transcriptional corepressor like 1 | 1.27 |
| *MCM5* | ENSG00000100297 | minichromosome maintenance complex component 5 | 1.24 |
| *SMC2* | ENSG00000136824 | structural maintenance of chromosomes 2 | 1.23 |
| *MIS18BP1* | ENSG00000129534 | MIS18 binding protein 1 | 1.22 |
| *CHAF1B* | ENSG00000159259 | chromatin assembly factor 1 subunit B | 1.21 |
| *MCM3* | ENSG00000112118 | minichromosome maintenance complex component 3 | 1.19 |
| *CDKN2C* | ENSG00000123080 | cyclin dependent kinase inhibitor 2C | 1.05 |
| *CABLES2* | ENSG00000149679 | Cdk5 and Abl enzyme substrate 2 | 1.04 |
| *MCM7* | ENSG00000166508 | minichromosome maintenance complex component 7 | 1.03 |
| *CDKN2A* | ENSG00000147889 | cyclin dependent kinase inhibitor 2A | -1 |
| *MAPK13* | ENSG00000156711 | mitogen-activated protein kinase 13 | -1.83 |
| *NEDD9* | ENSG00000111859 | neural precursor cell expressed, developmentally down-regulated 9 | -1.86 |
| *RGS2* | ENSG00000116741 | regulator of G protein signaling 2 | -1.96 |

**Supplementary Table S5. Up and down-regulated genes in positive regulation of cell proliferation process of FAM20A cells**

| **Gene names** | **Ensembl Gene ID** | **Description** | **Log2(fold-change)** |
| --- | --- | --- | --- |
| *HCK* | ENSG00000101336 | HCK proto-oncogene, Src family tyrosine kinase | -7.59 |
| *NTRK2* | ENSG00000148053 | neurotrophic receptor tyrosine kinase 2 | -5.48 |
| *BMP6* | ENSG00000153162 | bone morphogenetic protein 6 | -5.15 |
| *HTR2B* | ENSG00000135914 | 5-hydroxytryptamine receptor 2B | -4.95 |
| *HCLS1* | ENSG00000180353 | hematopoietic cell-specific Lyn substrate 1 | -3.78 |
| *CTSH* | ENSG00000103811 | cathepsin H | -3.7 |
| *BAMBI* | ENSG00000095739 | BMP and activin membrane bound inhibitor | -3.54 |
| *HAPLN3* | ENSG00000140511 | hyaluronan and proteoglycan link protein 3 | -2.61 |
| *ADRA2A* | ENSG00000150594 | adrenoceptor alpha 2A | -2.42 |
| *IGF2* | ENSG00000167244 | insulin like growth factor 2 | -1.83 |
| *FLT1* | ENSG00000102755 | fms related receptor tyrosine kinase 1 | -1.81 |
| *TBX2* | ENSG00000121068 | T-box transcription factor 2 | -1.76 |
| *ADM* | ENSG00000148926 | adrenomedullin | -1.68 |
| *ATF3* | ENSG00000162772 | activating transcription factor 3 | -1.68 |
| *LRP5* | ENSG00000162337 | LDL receptor related protein 5 | -1.5 |
| *FGF5* | ENSG00000138675 | fibroblast growth factor 5 | -1.25 |
| *BST1* | ENSG00000109743 | bone marrow stromal cell antigen 1 | -1.05 |
| *TNC* | ENSG00000041982 | tenascin C | 1.16 |
| *CDCA7L* | ENSG00000164649 | cell division cycle associated 7 like | 1.36 |
| *EZH2* | ENSG00000106462 | enhancer of zeste 2 polycomb repressive complex 2 subunit | 1.43 |
| *FBXO5* | ENSG00000112029 | F-box protein 5 | 1.57 |
| *FOXM1* | ENSG00000111206 | forkhead box M1 | 1.6 |
| *TBX3* | ENSG00000135111 | T-box transcription factor 3 | 1.66 |
| *KIF20B* | ENSG00000138182 | kinesin family member 20B | 1.91 |
| *AREG* | ENSG00000109321 | amphiregulin | 1.96 |
| *KIF14* | ENSG00000118193 | kinesin family member 14 | 2.4 |
| *ALDH3A1* | ENSG00000108602 | aldehyde dehydrogenase 3 family member A1 | 3.85 |
| *PRAME* | ENSG00000185686 | PRAME Nuclear Receptor Transcriptional Regulator | 10.6 |

**Supplementary Table S6. Up and down-regulated genes in the cell division process of FAM20A cells**

| **Gene names** | **Ensembl Gene ID** | **Description** | **Log2(fold-change)** |
| --- | --- | --- | --- |
| *KIF14* | ENSG00000118193 | kinesin family member 14 | 2.4 |
| *KNL1* | ENSG00000137812 | kinetochore scaffold 1 | 2.38 |
| *SKA1* | ENSG00000154839 | Spindle And Kinetochore Associated Complex Subunit 1 | 2.24 |
| *CENPF* | ENSG00000117724 | centromere protein F | 2.2 |
| *KIF18B* | ENSG00000186185 | kinesin family member 18B | 2.16 |
| *KIF11* | ENSG00000138160 | kinesin family member 11 | 2.06 |
| *NCAPH* | ENSG00000121152 | non-SMC condensin I complex subunit H | 2.03 |
| *GPSM2* | ENSG00000121957 | G protein signaling modulator 2 | 1.97 |
| *KIF20B* | ENSG00000138182 | kinesin family member 20B | 1.91 |
| *CCNA2* | ENSG00000145386 | cyclin A2 | 1.87 |
| *SPAG5* | ENSG00000076382 | sperm associated antigen 5 | 1.82 |
| *FAM83D* | ENSG00000101447 | family with sequence similarity 83 member D | 1.76 |
| *FBXO5* | ENSG00000112029 | F-box protein 5 | 1.57 |
| *NCAPG2* | ENSG00000146918 | non-SMC condensin II complex subunit G2 | 1.52 |
| *KNTC1* | ENSG00000184445 | kinetochore associated 1 | 1.52 |
| *TPX2* | ENSG00000088325 | TPX2 microtubule nucleation factor | 1.45 |
| *HELLS* | ENSG00000119969 | helicase, lymphoid specific | 1.43 |
| *CCNF* | ENSG00000162063 | cyclin F | 1.41 |
| *CDCA7L* | ENSG00000164649 | cell division cycle associated 7 like | 1.36 |
| *CDT1* | ENSG00000167513 | chromatin licensing and DNA replication factor 1 | 1.35 |
| *SMC4* | ENSG00000113810 | structural maintenance of chromosomes 4 | 1.33 |
| *MCM5* | ENSG00000100297 | minichromosome maintenance complex component 5 | 1.24 |
| *SMC2* | ENSG00000136824 | structural maintenance of chromosomes 2 | 1.23 |
| *MIS18BP1* | ENSG00000129534 | MIS18 binding protein 1 | 1.22 |
| *CDC6* | ENSG00000094804 | cell division cycle 6 | 1.19 |
| *MASTL* | ENSG00000120539 | microtubule associated serine/threonine kinase like | 1.13 |
| *NCAPD3* | ENSG00000151503 | non-SMC condensin II complex subunit D3 | 1.11 |
| *ZWILCH* | ENSG00000174442 | zwilch kinetochore protein | 1.04 |
| *CABLES2* | ENSG00000149679 | Cdk5 and Abl enzyme substrate 2 | 1.04 |
| *NCAPD2* | ENSG00000010292 | non-SMC condensin I complex subunit D2 | 1.02 |
| *NEDD9* | ENSG00000111859 | neural precursor cell expressed, developmentally down-regulated 9 | -1.86 |

**Supplementary Table S7. Up and down-regulated genes in apoptotic process of FAM20A cells**

| **Gene names** | **Ensembl Gene ID** | **Description** | **Log2(fold-change)** |
| --- | --- | --- | --- |
| *PRAME* | ENSG00000185686 | PRAME Nuclear Receptor Transcriptional Regulator | 10.6 |
| *RNF152* | ENSG00000176641 | ring finger protein 152 | 2.25 |
| *MAP2K6* | ENSG00000108984 | mitogen-activated protein kinase kinase 6 | 2.19 |
| *ESPL1* | ENSG00000135476 | extra spindle pole bodies like 1, separase | 1.87 |
| *TP63* | ENSG00000073282 | tumor protein p63 | 1.82 |
| *TPX2* | ENSG00000088325 | TPX2 microtubule nucleation factor | 1.45 |
| *HELLS* | ENSG00000119969 | helicase, lymphoid specific | 1.43 |
| *MCM2* | ENSG00000073111 | minichromosome maintenance complex component 2 | 1.37 |
| *MELK* | ENSG00000165304 | maternal embryonic leucine zipper kinase | 1.13 |
| *PHLDA1* | ENSG00000139289 | pleckstrin homology like domain family A member 1 | 1.09 |
| *CKAP2* | ENSG00000136108 | cytoskeleton associated protein 2 | 1.04 |
| *CDKN2A* | ENSG00000147889 | cyclin dependent kinase inhibitor 2A | -1 |
| *STK17A* | ENSG00000164543 | serine/threonine kinase 17a | -1.06 |
| *GJA1* | ENSG00000152661 | gap junction protein alpha 1 | -1.1 |
| *SMO* | ENSG00000128602 | smoothened, frizzled class receptor | -1.53 |
| *TBX2* | ENSG00000121068 | T-box transcription factor 2 | -1.76 |
| *SEMA6A* | ENSG00000092421 | semaphorin 6A | -2.08 |
| *TNFSF15* | ENSG00000181634 | TNF superfamily member 15 | -2.16 |
| *DAPK2* | ENSG00000035664 | death associated protein kinase 2 | -2.42 |
| *IGFBP3* | ENSG00000146674 | insulin like growth factor binding protein 3 | -2.68 |
| *PRUNE2* | ENSG00000106772 | prune homolog 2 with BCH domain | -2.81 |
| *SLC40A1* | ENSG00000138449 | solute carrier family 40 member 1 | -2.88 |
| *SERPINB9* | ENSG00000170542 | serpin family B member 9 | -3.03 |
| *DAPK1* | ENSG00000196730 | death associated protein kinase 1 | -3.07 |
| *CTSH* | ENSG00000103811 | cathepsin H | -3.7 |
| *GDF6* | ENSG00000156466 | growth differentiation factor 6 | -4.18 |
| *SULF1* | ENSG00000137573 | sulfatase 1 | -4.74 |


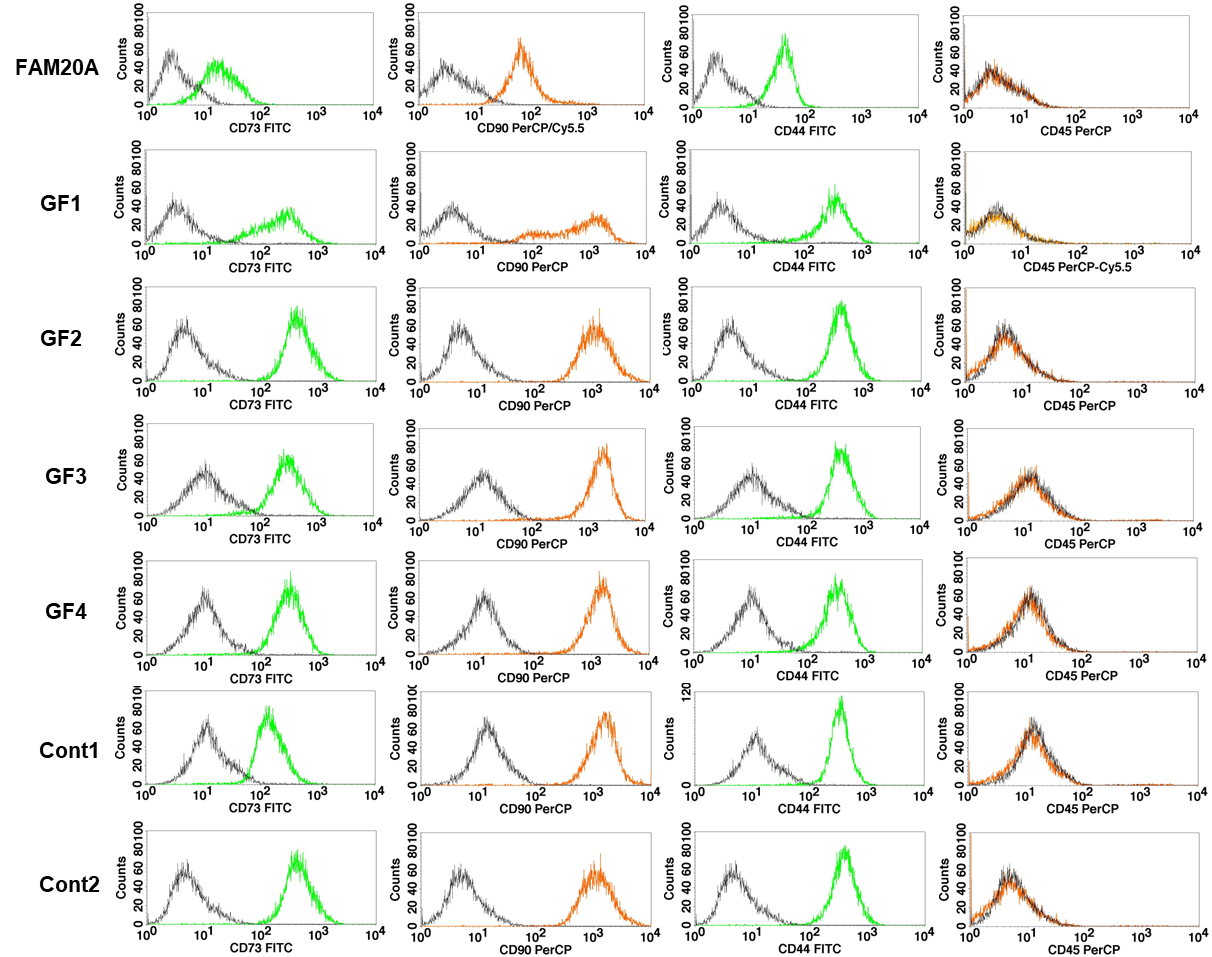


**Supplementary Figure S1. Expression of MSC cell surface markers in FAM20A cells and control gingival fibroblasts detected by flow cytometry.** GF1, GF2, GF3, and GF4 served as gingival fibroblast controls for RNA sequencing, while Cont1 and Cont2 were used as gingival fibroblast controls for cell experiments.


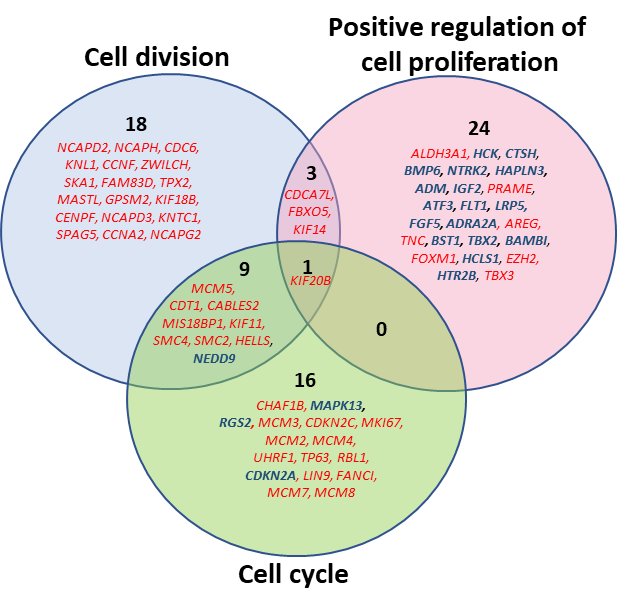


**Supplementary Figure S2. Venn diagram showing the overlap of DEGs across three processes: cell cycle, positive regulation of cell proliferation, and cell division.** Genes in red represent upregulated genes. Genes in blue indicate downregulated genes. The numbers in each section indicate the count of genes.
